# Low-dose atropine for myopia progression in children: a 2017–2024 systematic review and meta-analysis of randomized placebo-controlled trials

**DOI:** 10.3389/fmed.2026.1715033

**Published:** 2026-01-27

**Authors:** Luciano Magurno, Maximiliano Lang, Martina Zapata, Juan E. Gallo

**Affiliations:** 1 Instituto de Investigaciones en Medicina Traslacional (IIMT), Facultad de Ciencias Biomédicas, Universidad Austral, Consejo Nacional de Investigaciones Científicas y Técnicas (CONICET), Pilar/Buenos Aires, Argentina; 2 Departamento de Oftalmología, Hospital Universitario Austral, Pilar/Buenos Aires, Argentina

**Keywords:** atropine, axial length, children, meta-analysis, myopia, myopia control, randomized trial, refractive error

## Abstract

**Background:**

Low-dose atropine (0.01%) eye drops have emerged as a proposed intervention to slow myopic progression in children, but their short-term efficacy remains uncertain. We conducted a systematic review and meta-analysis of recent randomized, placebo-controlled trials to assess the efficacy and safety of 0.01% atropine in preventing myopia progression.

**Methods:**

A comprehensive search of PubMed, Embase, and Cochrane Library (through October 2024) identified double-blind randomized trials published from 2017 to 2024 that compared atropine 0.01% with placebo in children. Primary outcomes were the changes in refractive error (spherical equivalent) and axial length over at least 1 year. Secondary outcomes included treatment-related adverse events (e.g., photophobia). Data were extracted, pooled using random-effects models, and heterogeneity was assessed (I^2^ statistic).

**Results:**

Nine trials (*n* = 1,091) met inclusion. Spherical equivalent refraction (SER): MD +0.14 D/year (95% CI +0.04 to +0.24, *p* = 0.01; I^2^ = 64%). Axial length: MD −0.05 mm/year (95% CI −0.08 to −0.01, *p* = 0.01; I^2^ = 30%). Photophobia: RR 1.17 (95% CI 0.43–3.20, *p* = 0.69; I^2^ = 15%).

**Conclusion:**

At 12 months, 0.01% atropine yields small but statistically significant reductions in the progression of spherical-equivalent refraction and axial elongation, with no statistically significant increase in photophobia versus placebo, although absolute photophobia rates were higher in the atropine group (9.8% vs. 5.9%). Effects are modest and heterogeneous, and the 95% prediction intervals include the null, supporting the need for larger, longer-term trials to define the durability and clinical relevance of these findings.

**Systematic Review Registration:**

https://www.crd.york.ac.uk/prospero/, identifier CRD42024583729.

## 1 Introduction

Myopia is primarily the result of excessive axial elongation–most notably due to vitreous chamber lengthening–or, less commonly, to increased dioptric power of the eye’s refractive components (1). Axial length typically increases during childhood and may continue into adolescence and adulthood, potentially leading to high myopia (2) and, in severe cases, pathological myopia (3), one of the leading causes of irreversible visual impairment worldwide (4). The clinical concern is heightened by the sharp increase in the risk of pathological changes when refractive error reaches or exceeds −6 diopters (2–4).

Epidemiological projections highlight the scale of the problem. By 2050, myopia is expected to affect nearly 5 billion people globally, with high myopia projected to impact around 1 billion individuals (5). Given its significant contribution to visual disability (4) and its substantial impact on quality of life, there is a pressing need for effective interventions capable of slowing progression and reducing the risk of associated ocular complications.

Among the available options, pharmacological interventions–particularly topical atropine–have emerged as the most consistently effective. This antimuscarinic agent has demonstrated the ability to reduce myopia progression in children across multiple randomized clinical trials (6, 7). The 2011 Cochrane systematic review identified topical antimuscarinic medication as the most effective intervention for myopia control (8), and a 2020 update reaffirmed these findings, reporting strong evidence of clinically meaningful benefits.

Although the exact mechanism by which atropine slows myopia progression is not fully understood, experimental studies suggest that its effects may occur at the level of scleral remodeling. Myopia development is associated with marked biochemical and structural changes in the sclera–particularly in collagen architecture–that reduce its biomechanical integrity (9–11). Evidence shows increased extracellular matrix degradation and decreased synthesis of new matrix components in myopic eyes, making the sclera more prone to distension under physiological intraocular forces (12, 13). The identification of muscarinic acetylcholine receptor subtypes in human scleral fibroblasts supports the hypothesis that atropine may act directly on scleral tissue (14).

Therefore, this systematic review and meta-analysis aims to synthesize the most recent evidence from double-blind, placebo-controlled RCTs to provide a robust estimate of the 1-year efficacy and safety of 0.01% atropine in controlling myopia progression in children. We hypothesized that 0.01% atropine would demonstrate modest but statistically significant benefits in reducing refractive error progression and axial elongation, with a favorable safety profile, based on prior trial data. This design minimizes selection bias through randomization and reduces observer and participant influence via blinding and placebo comparison, providing a robust framework for evaluating the true therapeutic effect.

## 2 Materials and methods

### 2.1 Study design

We conducted a systematic review and meta-analysis in accordance with the Preferred Reporting Items for Systematic Reviews and Meta-Analyses (PRISMA) guidelines. The review protocol was registered on PROSPERO (CRD42024583729). The analysis was limited to previously published studies; no new clinical data were collected for this work.

### 2.2 Search strategy and selection criteria

A comprehensive literature search was performed in October 2024 using PubMed, Embase, and the Cochrane Library. The full search string for PubMed was: (myopia[MeSH Terms] OR myopia[Title/Abstract]) AND (atropine[MeSH Terms] OR atropine[Title/Abstract]) AND (child*[Title/Abstract] OR pediatric[Title/Abstract]) AND (randomized controlled trial[Publication Type] OR placebo[Title/Abstract]). Similar terms were adapted for Embase and Cochrane, including MeSH/Emtree. Complete, database-specific search strategies (including all field tags and limits) for PubMed, Embase, and Cochrane CENTRAL are provided in Supplementary Appendix S1. We also searched trial registries (ClinicalTrials.gov, WHO ICTRP) for unpublished trials, but none met inclusion. No language restrictions were applied; non-English studies (e.g., Chinese RCTs) were screened using translation tools if abstracts were available, but all included trials were in English. After removing duplicates, titles and abstracts were screened to exclude clearly irrelevant reports. The full text of remaining articles was then evaluated against the predefined inclusion criteria. We also hand-searched reference lists of relevant papers to identify any additional eligible studies.

### 2.3 Inclusion criteria

We included studies that: (1) were randomized, double-blind, placebo-controlled trials of topical atropine eye drops; (2) enrolled children roughly 4–15 years old with bilateral myopia (spherical equivalent refraction −0.50 D to −8.00 D in each eye, confirmed by cycloplegic refraction-studies with minor variations in baseline SER were included if groups were balanced at randomization); (3) tested a low concentration of atropine (defined here as 0.01% in at least one study arm); (4) had a follow-up duration of at least 1 year; and (5) reported on at least one of the primary efficacy outcomes (change in refractive error and/or change in axial length). Baseline axial length thresholds were not predefined, as they vary by age/ethnicity, but included studies had typical ranges (∼23–25 mm) with balanced groups. We required that trials also provide data on safety or side effects (particularly photophobia, given the known mydriatic effect of atropine). If a trial included multiple atropine concentrations, only data from the 0.01% atropine and placebo arms were considered for our primary analysis, in order to focus on the ultra-low dose.

### 2.4 Exclusion criteria

We excluded trials that combined atropine with other myopia control interventions (e.g., orthokeratology or multifocal lenses), studies in populations with coexisting ocular pathologies (e.g., strabismus, amblyopia) or prior myopia therapy, and studies with significant baseline differences between groups in age or refractive status (defined as *p* < 0.05 for between-group t-tests or equivalent on key variables like age, SER, AL). Non-randomized studies, open-label studies without a placebo control, and trials with follow-up shorter than 12 months were also excluded.

Two reviewers (L.M. and M.L.) independently screened all titles/abstracts and full-text articles for eligibility. Discrepancies were resolved by discussion with a third author (J.E.G.). In cases of multiple publications from the same trial, or an interim and final report, we included the most comprehensive or final dataset (to avoid double-counting patients).

### 2.5 Data extraction

Two reviewers independently extracted data using a standardized form; disagreements were resolved by consensus or, when needed, a third reviewer (J.E.G.). Extracted items included study design and setting, sample size, participant characteristics (age, ethnicity), atropine concentration and dosing frequency, follow-up duration, and outcomes.

### 2.6 Unit of analysis and handling of paired-eye outcomes

The unit of analysis was the child (participant). When outcomes were reported per eye, we extracted a single participant-level observation to avoid unit-of-analysis error due to within-child (inter-eye) correlation. We used the trial’s prespecified eye when stated (commonly the right eye). If a trial reported only a participant-level summary (e.g., mean of both eyes), we extracted that summary as reported. If outcomes were reported separately for right and left eyes without a prespecified eye or participant-level summary, we extracted the right-eye outcome to maintain a single observation per participant. We did not enter outcomes from both eyes of the same child as independent observations and therefore did not apply a paired-eye correlation correction or effective sample-size adjustment. If a trial analyzed both eyes using a method that accounts for within-child correlation (e.g., mixed models or generalized estimating equations) and reported a participant-level estimate, we extracted the reported estimate.

### 2.7 Outcome metrics and derived statistics

Primary outcomes were the change from baseline in spherical equivalent refraction (SER; diopters) and axial length (mm), analyzed as 12-months (annualized) change (D/year and mm/year). When a 12-months endpoint was available, we extracted the 12-months change; otherwise, we annualized the reported change over follow-up duration *T* years, assuming approximately linear change over time, by μ_yr_=μ_*T*_/*T*. Dispersion was scaled accordingly (*SD*_yr_ = *SD*_*T*_/*T*; SE_yr_ = SE_T_/T; equivalently, variances were scaled by 1 / *T*^2^). When multiple follow-up timepoints were reported, we used the timepoint closest to 12 months for the primary analysis. Secondary outcomes included adverse events, particularly photophobia. For studies reporting confidence intervals but not standard deviations, we derived SE from the 95% CI as SE = (*U* – *L*)/3.92 and applied the same annualization scaling when applicable. Reported effect estimates and 95% CIs were recorded when available; otherwise, they were calculated from extracted raw data. Tables 1, 2 summarize study characteristics and extracted quantitative findings.

**TABLE 1:** Characteristics of included randomized placebo-controlled trials (2017–2024).

References	Country	Atropine dose(s)	Follow-up	Mean age (years)	Ethnicity
Chan et al. (18)	China (HK)	0.01% vs. placebo	18 months	8.6 ± 1.0 (A); 8.4 ± 0.8 (P)	East Asian
Yam et al. (20)	China (HK)	0.05%, 0.025%, 0.01% vs. P	1 year	∼8.3 (range 6–12)^a^	East Asian
Wei et al. (21)	China	0.01% vs. placebo	1 year	9.6 ± 1.7 (combined)	East Asian
Hieda et al. (15)	Japan	0.01% vs. placebo	2 years	9.0 (median, combined)	East Asian
Saxena et al. (16)	India	0.01% vs. placebo	1 year	10.7 ± 2.2 (A); 10.8 ± 2.2 (P)	South Asian
Chia et al. (23)	Singapore	0.01%, 0.005%, 0.0025% vs. P	1 year	8.9 ± 1.3 (A 0.01%); 8.9 ± 1.7 (P)	East Asian
Hansen et al. (19)	Denmark	0.01%, 0.1% (loading) vs. P	1 year	9.4 ± 1.7 (combined)	European (Danish)
Repka et al. (22)	USA	0.01% vs. placebo	2 years	10.1 ± 1.8 (combined)	Multi-ethnic^b^
Sharma et al. (17)	India	0.01% vs. placebo	1 year	9.5 ± 2.6 (A); 9.7 ± 2.3 (P)	South Asian

A, atropine group; P, placebo group; HK, Hong Kong; loading, study included an initial higher dose phase (e.g., 0.1% for 6 months) followed by 0.01%.

*^a^*In Yam et al. (LAMP study), mean age by group: 0.05% (8.45 years), 0.025% (8.54), 0.01% (8.23), placebo (8.42).

*^b^*Repka et al. included 46% White, 18% East Asian, 15% Black, 15% Latino, 6% South Asian/other.

**TABLE 2:** Summary of outcomes in included trials: myopia progression over 1 year.

Study (atropine vs. placebo)	SER change (D) mean ± SD or (95% CI)	Axial length change (mm) mean ± SD or (95% CI)	Group difference (Atropine− Placebo)	*P*-value
Chan et al. (18) (0.01%)	−0.70 ± 0.39 vs. −0.66 ± 0.41	+0.32 ± 0.16 vs. +0.30 ± 0.22	−0.03 D/year (95% CI −0.16 to 0.11); +0.01 mm/year (−0.05 to 0.08)	0.63 0.52
Yam et al. (20) (LAMP 0.01%)	−0.59 (0.61) D/year vs. −0.81 (0.53) D/year^a^	+0.36 (0.29) mm/year vs. +0.41 (0.22) mm/year	+0.22 D/year (0.07 to 0.37); −0.05 mm/year (−0.12 to 0.02)	<0.001 0.18
Wei et al. (21) (0.01%)	−0.49 ± 0.42 vs. −0.76 ± 0.50	+0.32 ± 0.19 vs. +0.41 ± 0.19	+0.27 D/year (95% CI 0.13 to 0.41); −0.09 mm/year (95% CI −0.15 to −0.03)	<0.001 0.004
Hieda et al. (15) (0.01%)	−0.63 (−0.68, −0.59) vs. −0.74 (−0.79, −0.70)^b^	+0.32 (0.30, 0.34) vs. +0.39 (0.37, 0.41)	+0.08 D/year (95% CI −0.05 to 0.21); −0.04 mm/year (95% CI −0.10 to 0.02)	<0.001 <0.001
Saxena et al. (16) (0.01%)	−0.16 ± 0.40 vs. −0.35 ± 0.40	+0.22 ± 0.20 vs. +0.28 ± 0.28	+0.19 D/year (0.03 to 0.35); −0.06 mm/year (−0.16 to 0.04)	0.021 0.19
Chia et al. (23) (0.01%)	−0.31 ± 0.29 vs. −0.80 ± 0.66 (per year)	+0.14 ± 0.09 vs. +0.39 ± 0.14 (per year)	+0.25 D/year (0.00 to 0.50); −0.11 mm/year (−0.23 to −0.01)	0.0056 0.0026
Hansen et al. (19) (0.01%)	−0.51 ± 1.13 vs. −0.60 ± 1.04^c^	+0.07 ± 0.78 vs. +0.26 ± 0.90^c^	+0.19 D/year (95% CI 0.00 to 0.38); −0.07 mm/year (95% CI −0.15 to 0.01)	0.14 0.02
Repka et al. (22) (0.01%)	−0.41 (−0.48 to −0.34) vs. −0.40 (−0.49 to −0.31)^b^	+0.22 (0.20 to 0.25) vs. +0.23 (0.19 to 0.26)	–0.01 D/year (95% CI −0.10 to +0.08); 0.00 mm/year (95% CI −0.05 to +0.05)	0.83 0.83
Sharma et al. (17) (0.01%)	−0.31 ± 0.50 vs. −0.80 ± 1.65	+0.11 ± 0.22 vs. +0.23 ± 0.44	+0.49 D/year (0.01 to 0.97); −0.12 mm/year (−0.26 to 0.02)	0.003 0.007

SER, spherical equivalent refraction; D, diopters; SD, standard deviation; CI, confidence interval. Positive differences indicate less myopia progression (or less axial elongation) with atropine.

*^a^*Yam et al. (20) LAMP study reported 1-year changes for multiple atropine concentrations; here 0.01% vs. placebo annualized from published data.

*^b^*Hieda et al. (15) and Repka et al. (22) reported 2-year changes; values are annualized to 1-year by dividing mean changes and their corresponding CI limits by 2.

*^c^*Hansen et al. (19) had two atropine arms (0.01% and 0.1% loading dose); the 1-year placebo vs. 0.01% comparison is shown.

### 2.8 Risk of bias assessment

The risk of bias in each trial was assessed using the Cochrane Risk of Bias 2 (RoB 2) tool for randomized trials. This evaluates potential bias across five domains: (1) randomization process, (2) deviations from intended interventions, (3) missing outcome data, (4) outcome measurement, and (5) selective reporting. Two reviewers performed the bias assessments independently, with any disagreements resolved by consensus. Each trial was rated as “low risk,” “some concerns,” or “high risk” of bias on each domain and overall. Table 3 provides the full domain-level assessments for each study. In our cohort, all trials were blinded and placebo-controlled, minimizing performance and detection biases. No trial was judged to have a high overall risk of bias. Most were rated low risk across domains; however, five trials had some concerns in at least one domain [Hieda et al. (15): missing outcome data; Saxena et al. (16): randomization, missing outcome data, selection of the reported result; Sharma et al. (17): randomization, deviations, missing outcome data, selection of the reported result; Chan et al. (18) and Hansen et al. (19): selection of the reported result]. These methodological quality findings are noted when interpreting the results. A more complete explanation of each RoB2 domain for each of the studies can be found in Supplementary Appendix S1.

**TABLE 3:** Risk of bias summary for randomized studies (RoB 2).

References	Bias from randomization process	Bias due to deviations from intended interventions	Bias due to missing outcome data	Bias in measurement of the outcomes	Bias in selection of the reported result	Overall risk of bias
Yam et al. (20)	Low	Low	Low	Low	Low	Low
Wei et al. (21)	Low	Low	Low	Low	Low	Low
Hieda et al. (15)	Low	Low	Some concerns	Low	Low	Some concerns
Saxena et al. (16)	Some concerns	Low	Some concerns	Low	Some concerns	Some concerns
Repka et al. (22)	Low	Low	Low	Low	Low	Low
Hansen et al. (19)	Low	Low	Low	Low	Some concerns	Some concerns
Chia et al. (23)	Low	Low	Low	Low	Low	Low
Sharma et al. (17)	Some concerns	Some concerns	Some concerns	Low	Some concerns	Some concerns
Chan et al. (18)	Low	Low	Low	Low	Some concerns	Some concerns

Sterne et al. (28). Green shading indicates “low risk of bias”; yellow shading indicates ‘some concerns”.

### 2.9 Statistical analysis

All meta-analyses were performed using Review Manager web version 9.17.0. Random-effects models were used throughout with inverse-variance weighting. For continuous outcomes (spherical equivalent refraction [D/year] and axial length [mm/year]), effects were pooled as mean differences (atropine − placebo) with 95% confidence intervals calculated using the Hartung–Knapp–Sidik–Jonkman (HKSJ) method, and between-study variance (τ^2^) estimated using restricted maximum likelihood (REML). For dichotomous outcomes (photophobia), effects were pooled as risk ratios with 95% confidence intervals calculated using HKSJ, and τ^2^; estimated using the DerSimonian–Laird method, consistent with the analysis settings used for that outcome. Statistical heterogeneity was assessed using Cochran’s Q and quantified with I^2^. Prediction intervals were calculated for random-effects models to reflect the expected range of effects in a future similar study. A two-sided *P* < 0.05 was considered statistically significant.

## 3 Results

### 3.1 Study selection

**FIGURE 1:**
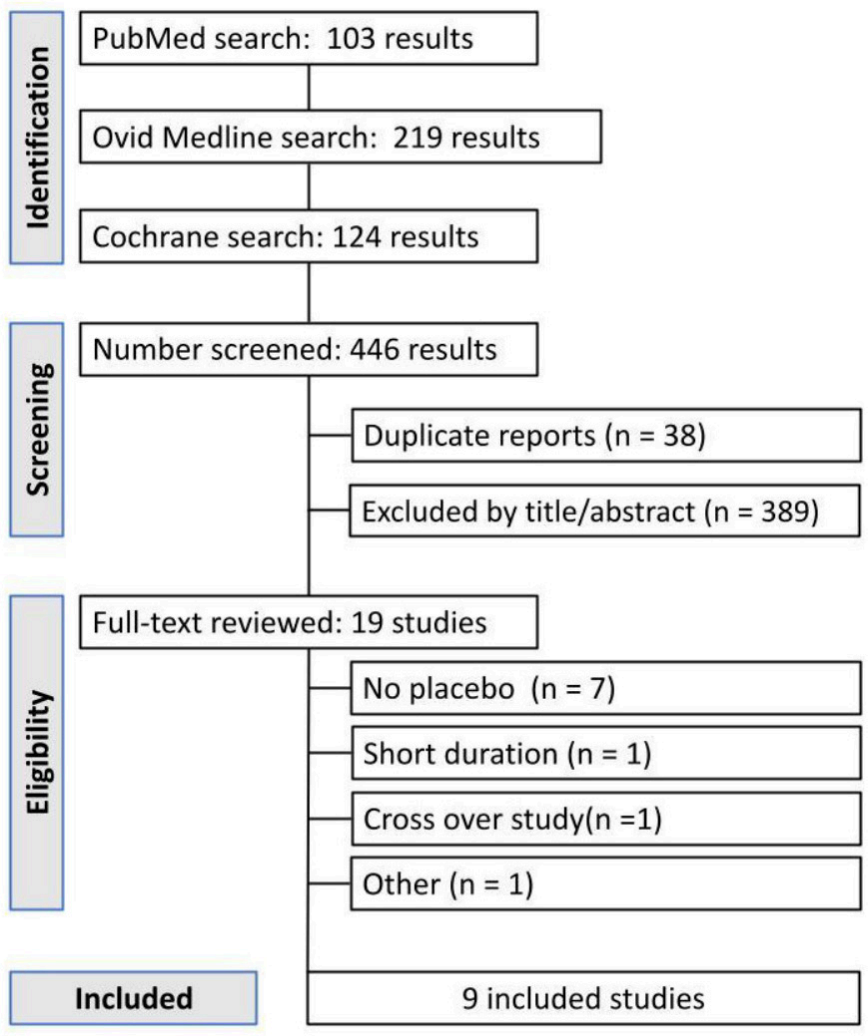
PRISMA flow diagram of study selection.

The literature search yielded 312 unique records. After title/abstract screening, 27 full-text articles were reviewed. Of these, 18 were excluded (Figure 1 provides details of exclusions, such as ineligible study design or insufficient follow-up), leaving 9 randomized controlled trials (15–23) for inclusion in this review (Figure 1). These 9 trials were published between 2017 and 2024 and comprised a total of 1091 pediatric participants (576 in atropine 0.01% groups and 515 in placebo groups). Table 1 summarizes the key characteristics of the included studies. All trials were double-blind and placebo-controlled, and all but one were single-center studies (the exception being a multicenter trial in India). Study sample sizes ranged from 60 to 400 children, with follow-up durations of 1 year in most cases (one trial followed participants for 18 months, which we annualized for comparison). Geographically, the studies were conducted in East Asia (Hong Kong, mainland China, Japan), South Asia (India), Europe (Denmark), and the United States.

### 3.2 Baseline characteristics

The mean age of participants at enrollment ranged from 8 to 10 years across trials, with most studies having slightly more female than male participants. Baseline refractive error and axial length were generally similar between the atropine and placebo groups within each study (mean spherical equivalent around −2 to −3 D, and mean axial length roughly 24 mm, with some variation by population). Ethnic composition varied: the U.S. trial included a multi-ethnic cohort (46% White, 18% East Asian, 15% Black, 15% Latino, 6% other), the Danish trial involved European Caucasian children, and the Asian trials predominantly involved East or South Asian children. These differences are noted because ethnicity can influence both myopia progression rates and treatment response.

### 3.3 Primary outcomes

**FIGURE 2:**
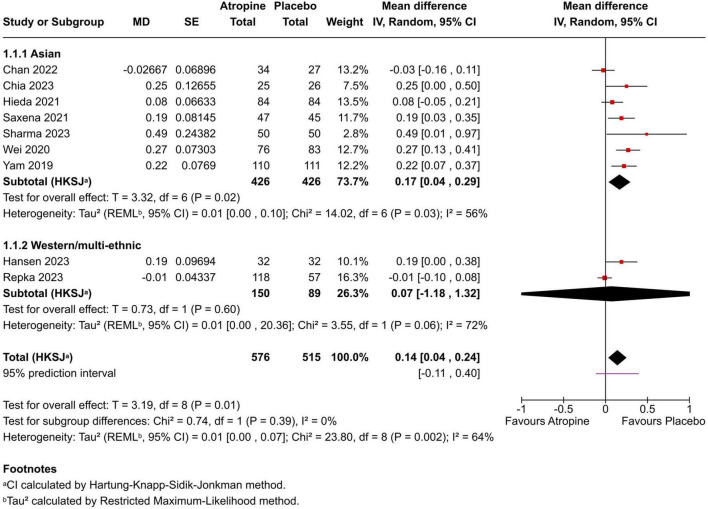
Forest plot of mean change in refractive error (spherical equivalent) in diopters (D) over 1 year, comparing 0.01% atropine vs. placebo. Negative values indicate myopic progression (increase in myopia). Squares represent individual study mean differences (atropine minus placebo) proportional to study weight; the diamond represents the pooled estimate (random-effects model).

Change in spherical-equivalent refraction (SER): Effects are expressed as mean difference (MD, atropine − placebo) in diopters per 12 months, where positive values favor atropine (less myopic shift). The pooled MD was +0.14 D/year (95% CI +0.04 to +0.24; *p* = 0.01; I^2^ = 64%) using REML with Hartung–Knapp adjustment (Figure 2). Seven of nine studies showed point estimates favoring atropine; one was essentially null and one favored placebo. The 95% prediction interval was −0.11 to +0.40 D/year.

**FIGURE 3:**
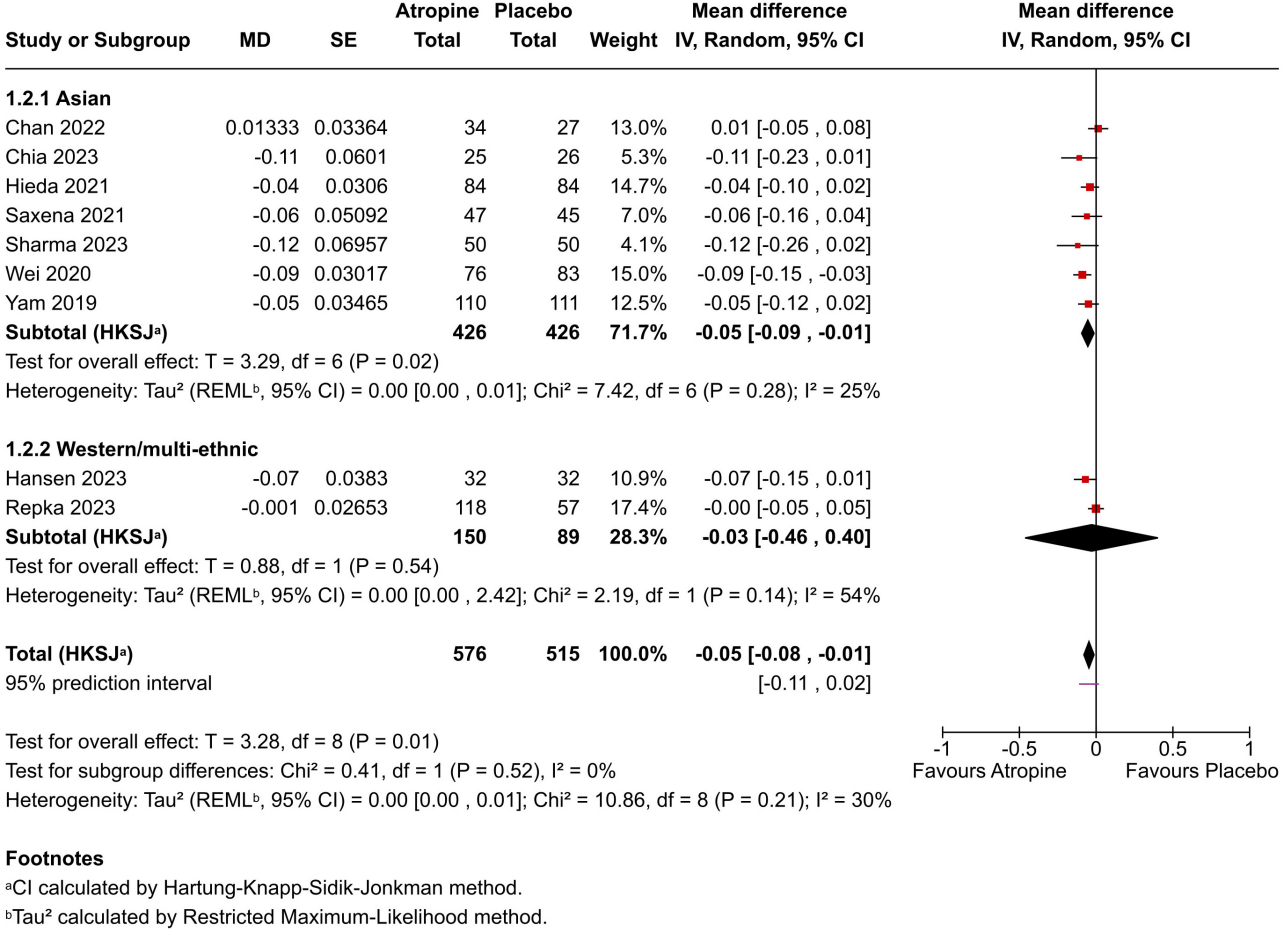
Forest plot of mean change in axial length (mm/year) comparing 0.01% atropine vs. placebo. Negative mean differences indicate less axial elongation in the atropine group.

Change in axial length (AL): Effects are expressed as MD (atropine − placebo) in millimeters per 12 months, where negative values favor atropine (less axial elongation). The pooled MD was −0.05 mm/year (95% CI −0.08 to −0.01; *p* = 0.01; I^2^ = 30%) (Figure 3). Most trials showed small reductions in axial elongation with atropine; one was essentially null and one slightly favored placebo. The 95% prediction interval was −0.11 to +0.02 mm/year.

### 3.4 Safety outcome

**FIGURE 4:**
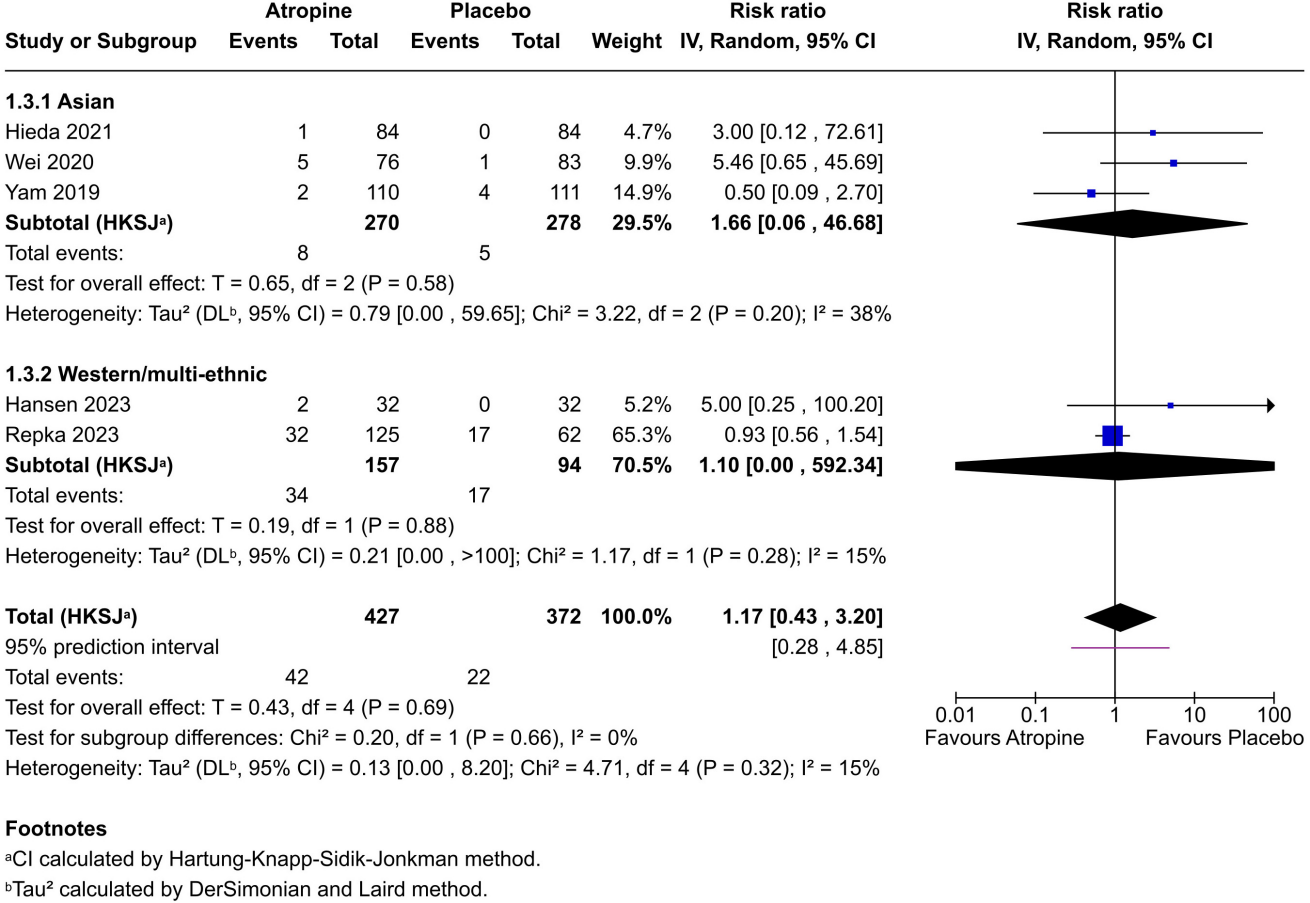
Forest plot of risk ratio for photophobia (light sensitivity) with 0.01% atropine vs. placebo. A risk ratio above 1 indicates higher risk in the atropine group, but here the estimate is not significant. Trials with zero events in both arms were excluded from RR pooling; photophobia was meta-analyzed only in studies reporting ≥1 event in at least one arm.

Five trials contributed dichotomous safety data for photophobia, with definitions varying (e.g., self-reported light sensitivity vs. clinical grading); this and potential for selective reporting may bias estimates. The pooled risk ratio (RR) for photophobia with 0.01% atropine vs. placebo was 1.17 (95% CI 0.43–3.20; *p* = 0.69; I^2^ = 15%) with a 95% prediction interval 0.28–4.85 (Figure 4). Absolute event counts were 42/427 (9.8%) in atropine and 22/372 (5.9%) in placebo arms. Overall, 0.01% atropine did not increase photophobia risk compared with placebo, and reported events were generally mild. Other adverse effects (e.g., accommodation difficulty, near-vision symptoms, pupil changes) were assessed inconsistently across trials, limiting synthesis.

### 3.5 Sub-group analysis by region/ethnicity

To explore heterogeneity, we conducted sub-group analyses by region/ethnicity: Asian cohorts (7 trials: Chan, Yam, Wei, Hieda, Saxena, Chia, Sharma; *n* = 852) vs. Western/multi-ethnic (2 trials: Hansen [European], Repka [multi-ethnic US]; *n* = 239). For SER, the pooled MD was +0.17 D/year (95% CI +0.04 to +0.29; *p* < 0.03; I^2^ = 56%) in Asian groups vs. +0.07 D/year (95% CI −1.18 to +1.32; *p* = 0.06; I^2^ = 72%) in Western/multi-ethnic. For AL, −0.05 mm/year (95% CI −0.09 to −0.01; *p* < 0.28; I^2^ = 25%) in Asian vs. −0.03 mm/year (95% CI −0.46 to +0.40; *p* = 0.14; I^2^ = 54%) in Western/multi-ethnic. Test for sub-group differences: *p* = 0.39 for SER, *p* = 0.52 for AL. These suggest stronger effects in Asian populations, potentially due to faster baseline progression rates. However, limited studies per sub-group and inability to analyze other factors (e.g., age, baseline SER) restrict generalizability.

### 3.6 Quality assessment

The individual risk-of-bias assessment for the nine included randomized controlled trials is summarized in Table 3. The evaluation was performed using the revised Cochrane risk-of-bias tool for randomized trials (RoB 2.0). Five trials [Yam et al. (20), Wei et al. (21), Repka et al. (22), Hansen et al. (19), and Chia et al. (23)] were judged to have an overall low risk of bias across all domains. In contrast, four studies–Hieda et al. (15), Saxena et al. (16), Sharma et al. (17), and Chan et al. (18)–were rated as having “some concerns.” These concerns were mainly related to missing outcome data (Hieda, Saxena, Sharma), the randomization process (Saxena, Sharma), deviations from intended interventions (Sharma), and selective reporting of results (Saxena, Sharma, Chan). Importantly, no trial was assessed as having a high risk of bias in any domain.

## 4 Discussion

In this meta-analysis of nine randomized, double-masked, placebo-controlled trials of 0.01% atropine, we found small but statistically significant 1-year effects on both spherical equivalent refraction (SER) and axial length (AL), accompanied by a favorable safety profile. The pooled SER effect was +0.14 D/year (95% CI +0.04 to +0.24; *p* = 0.01; I^2^ = 64%) using REML with Hartung–Knapp adjustment, and the pooled AL effect was −0.05 mm/year (95% CI −0.08 to −0.01; *p* = 0.01; I^2^ = 30%). These magnitudes indicate that, at 0.01%, atropine confers modest average benefits over 12 months. However, the 95% prediction intervals for both outcomes (−0.11 to +0.40 D/year for SER; −0.11 to +0.02 mm/year for AL) include the null, underscoring that future, similar trials could observe no effect, and highlighting between-study heterogeneity, particularly for SER. Clinically, these effects may not be meaningful for all patients, especially slower progressors. Although statistically significant, the pooled effects were small (SER 0.14 D/year; AL −0.05 mm/year) and may fall below pragmatic thresholds for a minimal clinically important difference (MCID) (e.g., ≥0.25 D/year slowing in SER or ≥0.1 mm/year slowing in axial length). Formal MCIDs for SER and axial length are not universally established; therefore, these are presented as pragmatic benchmarks for clinical context.

Trial-level patterns help contextualize these pooled estimates. In placebo arms, annual myopic progression ranged from roughly −0.8 D/year to −1.5 D/year, typically faster in East Asian cohorts. In 0.01% atropine arms, progression ranged from about −0.3 D/year to −1.4 D/year. Two trials–Wei et al. (21) (China) and Sharma et al. (17) (India)–reported statistically significant slowing of SER progression with 0.01% atropine on the order of ∼0.25–0.50 D less myopia at 1 year (*P* < 0.01) (22), though clinically modest; several others showed smaller, non-significant differences. For AL, Wei et al. (21), Hieda et al. (15), and Hansen et al. (19) observed significantly less axial elongation with atropine (approximately 0.10–0.20 mm over 1 year), consistent with our pooled −0.05 mm/year estimate. By contrast, the two largest and most recent 1-year trials (U.S. and Denmark) reported no meaningful between-group differences in either SER or AL (*P* > 0.8), reinforcing that any 1-year effect at 0.01% is modest and context-dependent.

The joint significance of SER and AL at one year lessens the previously noted SER–AL “dissociation,” yet differences in measurement properties and susceptibility to bias remain relevant. AL is a more objective and stable biomarker than SER, which can be influenced by cycloplegia protocols, lenticular changes, and chorio-retinal compensation. Variability in biometry devices (e.g., IOLMaster vs. ultrasound) and cycloplegia (e.g., tropicamide vs. cyclopentolate) across trials may contribute to outcome variability. It is biologically plausible that anatomical changes precede refractive changes, such that >1 year of continuous therapy may be required for small AL differences to translate into clinically perceptible SER benefits. This interpretation accords with longer-duration evidence, including ATOM2 (24), the 2-years Japanese trial (15), and the Danish trial (19), all of which suggest that benefits may require prolonged therapy, consistent with slow scleral-remodeling mechanisms at low dose.

Sources of heterogeneity likely include baseline progression rate, age, and ethnic composition–with East Asian, younger, faster-progressing cohorts showing larger absolute gains–alongside environmental exposures (outdoor time, near work) that were inconsistently measured and rarely standardized. Increased time spent outdoors at school has been shown to reduce the development of myopia in children (25). Methodological differences (cycloplegia, biometry devices, sample size, follow-up, adherence, and formulation/compounding) can dilute or inflate effects, particularly for SER (I^2^ = 64%, moderate-high, implying ∼64% of variability is true heterogeneity vs. sampling error; implications: pooled estimates less reliable for prediction). In parallel, the well-described dose–response from programs such as LAMP (20) and APPLE (23)–greater efficacy at higher concentrations (e.g., 0.05% or 1%) at the expense of tolerability–positions 0.01% at the maximal-tolerability/minimal-efficacy end of the short-term trade-off. Suggestions for intermediate doses (e.g., 0.025%–0.05%) are based on external evidence, not this meta-analysis.

Safety findings were reassuring. Across five trials with analyzable data, photophobia did not differ significantly between 0.01% atropine and placebo (RR 1.17, 95% CI 0.43–3.20; *p* = 0.69; I^2^ = 15%; 95% prediction interval 0.28–4.85), with no statistically significant increase despite numerically higher absolute counts in atropine arms (42/427 [9.8%] vs. 22/372 [5.9%]). Event definitions varied across trials, and most reported episodes were mild; several studies recorded zero clinically relevant cases in either arm. Other adverse events–including near-work difficulty related to accommodation, allergic conjunctivitis, and blurred vision–were infrequent and similar to placebo at 0.01% (26). At low dose, atropine remains well tolerated, which is a key advantage when counseling families.

Ethnic/regional differences: as shown in sub-group analyses, effects were stronger in Asian cohorts (SER +0.17 D/year, AL −0.05 mm/year) vs. Western/multi-ethnic (SER +0.07 D/year, AL −0.03 mm/year), aligning with faster baseline progression in East Asian children (e.g., −0.8 to −1.5 D/year placebo) per Jones-Jordan (27). This may reflect genetic/environmental factors (e.g., outdoor time, near work), emphasizing context-dependent efficacy. Western trials (Hansen, Repka) showed no meaningful differences, suggesting limited benefit in slower-progressing populations.

This review has strengths, including restriction to placebo-controlled RCTs, the use of random-effects models with Hartung–Knapp adjustment, and parallel synthesis of efficacy and safety. Limitations include heterogeneous methods and reporting, incomplete standardization of environmental covariates, and limited ability to explore small-study effects or conduct robust subgroup meta-analyses (e.g., by ethnicity or baseline SER)–these restrict generalizability and should be addressed in future work. The fact that prediction intervals encompass the null for both primary outcomes cautions that average effects may not be consistently reproducible across settings, particularly over a 1-year horizon. Prediction intervals estimate the range where effects of future similar studies might fall, implying real-world variability beyond the CI.

Clinically, these results support shared decision-making. For families prioritizing tolerability, 0.01% atropine offers excellent safety with small average benefits on SER and AL at 1 year. In faster progressors or when a more pronounced 1-year effect is desired, higher doses may be considered based on external data, accepting higher rates of photophobia and accommodation symptoms. Decisions should integrate pace of progression, age, ethnicity, outdoor/near-work behaviors, and family tolerance for adverse effects.

Future research should prioritize adequately powered, longer-duration RCTs (≥2–3 years) with head-to-head dose-ranging comparisons (0.01/0.025/0.05%), ethnically diverse cohorts with prespecified subgroup analyses, centralized biometry, and standardized environmental and cycloplegia protocols, while reporting patient-important outcomes (transition to high myopia, need for rescue therapy, quality of life) alongside SER and AL. Additionally, define standardized outcome measures and MCIDs (e.g., ≥0.25 D/year SER or ≥0.1 mm/year AL reduction) for myopia control. In sum, at 0.01%, atropine is safe and yields small, statistically significant reductions in progression of SER and AL at 12 months on average, yet heterogeneity and prediction intervals crossing the null indicate that effects are modest, context-dependent, and not uniformly reproducible across settings–reinforcing the need for longer, dose-ranging trials to define its optimal role in myopia control.

## Data Availability

The original contributions presented in this study are included in this article/Supplementary material, further inquiries can be directed to the corresponding author.
