# Supplementary material for: Low-dose atropine for myopia progression in children: a 2017–2024 systematic review and meta-analysis of randomized placebo-controlled trials
Source: Front Med (Lausanne). 2026 Jan 27;13:1715033. doi: 10.3389/fmed.2026.1715033 (PMC12886047; doi:10.3389/fmed.2026.1715033)
Supplement: Supplementary file 1 [file Supplementary_file_1.docx]

"Low-Dose Atropine for Myopia Progression in Children: A 2017–2024 Systematic Review and Meta-Analysis of Randomized Placebo-Controlled Trials"

SUPPLEMENTARY APPENDIX S1

*Detailed Search Strategies*

A) Detailed Search Strategies (October 2024)

A1) PubMed (MEDLINE; National Library of Medicine)

- Platform: PubMed

- Date last searched: October 2024

- Search string:

(myopia[MeSH Terms] OR myopia[Title/Abstract]) AND

(atropine[MeSH Terms] OR atropine[Title/Abstract]) AND

(child*[Title/Abstract] OR pediatric[Title/Abstract]) AND

(randomized controlled trial[Publication Type] OR placebo[Title/Abstract])

Limits/filters:

- No language restrictions

- No date restrictions

- Human/age filters: none applied

A2) Embase (Elsevier)

- Platform: Embase.com

- Date last searched: October 2024

- Search string:

('myopia'/exp OR myopia:ti,ab) AND

('atropine'/exp OR atropine:ti,ab) AND

(child*:ti,ab OR pediatric:ti,ab) AND

('randomized controlled trial'/de OR placebo:ti,ab)

Limits/filters:

- No language restrictions

- No date restrictions

A3) Cochrane CENTRAL (Cochrane Library)

- Platform: Cochrane Library – CENTRAL

- Date last searched: October 2024

- Search string:

(myopia):ti,ab,kw AND

(atropine):ti,ab,kw AND

(child* OR pediatric):ti,ab,kw AND

(random* OR placebo):ti,ab,kw

Limits/filters:

- No language restrictions

- No date restrictions

*Risk of Bias (RoB 2) Assessments*

B) Risk of Bias (RoB 2) – Domain-level assessments and brief justifications

B1) RoB 2 assessment approach

Risk of bias was assessed using the Cochrane Risk of Bias 2 (RoB 2) tool across five domains:

(1) randomization process,

(2) deviations from intended interventions,

(3) missing outcome data,

(4) measurement of the outcome,

(5) selection of the reported result.

Two reviewers performed assessments independently, resolving disagreements by consensus.

B2) Table S1. RoB 2 domain-level judgments (by trial)

| Study | Bias from randomization process | Bias due to deviations from intended interventions | Bias due to missing outcome data | Bias in measurement of the outcomes | Bias in selection of the reported result | Overall risk of bias |
| --- | --- | --- | --- | --- | --- | --- |
| Yam 2019 | Low | Low | Low | Low | Low | Low |
| Wei 2020 | Low | Low | Low | Low | Low | Low |
| Hieda 2021 | Low | Low | Some Concerns | Low | Low | Some Concerns |
| Saxena 2021 | Some Concerns | Low | Some Concerns | Low | Some Concerns | Some Concerns |
| Repka 2023 | Low | Low | Low | Low | Low | Low |
| Hansen 2023 | Low | Low | Low | Low | Some Concerns | Some Concerns |
| Chia 2023 | Low | Low | Low | Low | Low | Low |
| Sharma 2023 | Some Concerns | Some Concerns | Some Concerns | Low | Some Concerns | Some Concerns |
| Chan 2022 | Low | Low | Low | Low | Some Concerns | Some Concerns |

B3) Table S2. Justifications for RoB 2 judgments (focused on domains with “Some concerns”)

Yam 2019 — Low risk across domains.

- No important concerns identified in RoB 2 domains based on the trial report.

Wei 2020 — Low risk across domains.

- No important concerns identified in RoB 2 domains based on the trial report.

Hieda 2021 — Some concerns (Missing outcome data).

- Missing outcome data: Some concerns due to loss to follow-up and/or incomplete outcome reporting across follow-up, with limited information to confirm missingness was unlikely to bias results materially.

Saxena 2021 — Some concerns (Randomization; Missing outcome data; Selective reporting).

- Randomization: Some concerns because allocation concealment and/or random sequence details were insufficiently reported to fully rule out baseline imbalance risk.

- Missing outcome data: Some concerns due to attrition and limited information demonstrating missingness was balanced and unlikely to bias estimates.

- Selective reporting: Some concerns because prespecification of outcomes/timepoints and analysis plan could not be confirmed clearly from publicly available protocol/registry information in the report.

Repka 2023 — Low risk across domains.

- No important concerns identified in RoB 2 domains based on the trial report.

Hansen 2023 — Some concerns (Selective reporting).

- Selective reporting: Some concerns because prespecification (protocol/registration/analysis plan) for the reported outcomes/timepoints was not clearly verifiable from the publication, limiting certainty that results were free from selective reporting.

Chia 2023 — Low risk across domains.

- No important concerns identified in RoB 2 domains based on the trial report.

Sharma 2023 — Some concerns (Randomization; Deviations; Missing outcome data; Selective reporting).

- Randomization: Some concerns due to unclear reporting of random sequence generation and/or allocation concealment.

- Deviations: Some concerns because information on adherence/co-interventions and how deviations were handled analytically was limited.

- Missing outcome data: Some concerns due to incomplete follow-up and limited evidence that missingness would not bias effect estimates.

- Selective reporting: Some concerns because prespecified outcomes/timepoints could not be clearly confirmed.

Chan 2022 — Some concerns (Selective reporting).

- Selective reporting: Some concerns because prespecification of outcomes/timepoints and/or statistical analysis plan was not clearly verifiable from the publication, limiting certainty about selective reporting risk.
